# Supplementary material for: Neutrophil extracellular traps-inspired DNA hydrogel for wound hemostatic adjuvant
Source: Nat Commun. 2024 Jul 2;15:5557. doi: 10.1038/s41467-024-49933-3 (PMC11219873; doi:10.1038/s41467-024-49933-3)
Supplement: Supplementary file 1 — Supplementary Information [file 41467_2024_49933_MOESM1_ESM.docx]

# Supplementary Information

# Neutrophil Extracellular Traps-inspired DNA Hydrogel for Wound Hemostatic Adjuvant

Rui Ye^1^#, Ziyu Zhu^2,3^#, Tianyi Gu^2^#, Dengjie Cao^1^, Kai Jiang^1^, Qiang Dai^4,5^, Kuoran Xing^3^, Yifan Jiang^3,6^, Siyi Zhou^2^, Ping Cai^1^, David Tai Leong^3^*, Mengfei Yu^2^*, Jie Song^1,4^*

^1^Institute of Nano Biomedicine and Engineering, Department of Instrument Science and Engineering, School of Electronic Information and Electrical Engineering, Shanghai Jiao Tong University, Shanghai 200240, China.

^2^The Affiliated Hospital of Stomatology, School of Stomatology, Zhejiang University School of Medicine, and Key Laboratory of Oral Biomedical Research of Zhejiang Province, Hangzhou, Zhejiang 310006, China.

^3^Department of Chemical and Biomolecular Engineering, National University of Singapore, 4 Engineering Drive 4, Singapore 117585, Singapore.

^4^Hangzhou Institute of Medicine, Chinese Academy of Sciences, Hangzhou, Zhejiang 310022, China.

^5^College of Materials Science and Engineering, Zhejiang University of Technology, Hangzhou, Zhejiang 310014, China.

^6^Department of Ultrasound in Medicine, The Second Affiliated Hospital of Zhejiang University School of Medicine, Zhejiang University, Hangzhou 310009, China.

#These authors equally contributed to this work.

*Email addresses for correspondence: [sjie@sjtu.edu.cn](mailto:sjie@sjtu.edu.cn);

[yumengfei@zju.edu.cn](mailto:yumengfei@zju.edu.cn);

cheltwd@nus.edu.sg

**Supplementary Figures**


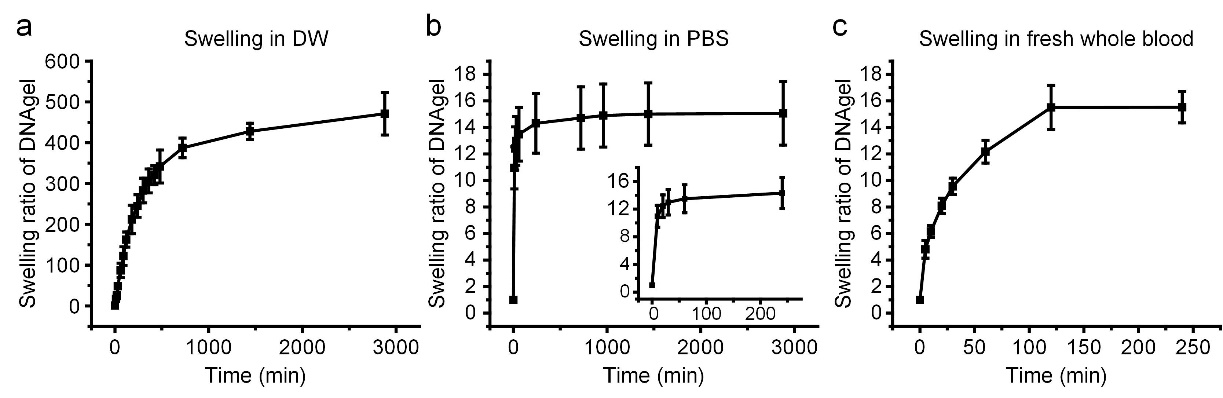


**Supplementary Figure 1.** Swelling ratio of DNAgel. DNAgel swelled in (**a**) DW, (**b**) PBS, and (**c**) fresh whole blood. Data were presented as mean ± SD (*n* = 3). Source data are provided as a Source Data file.


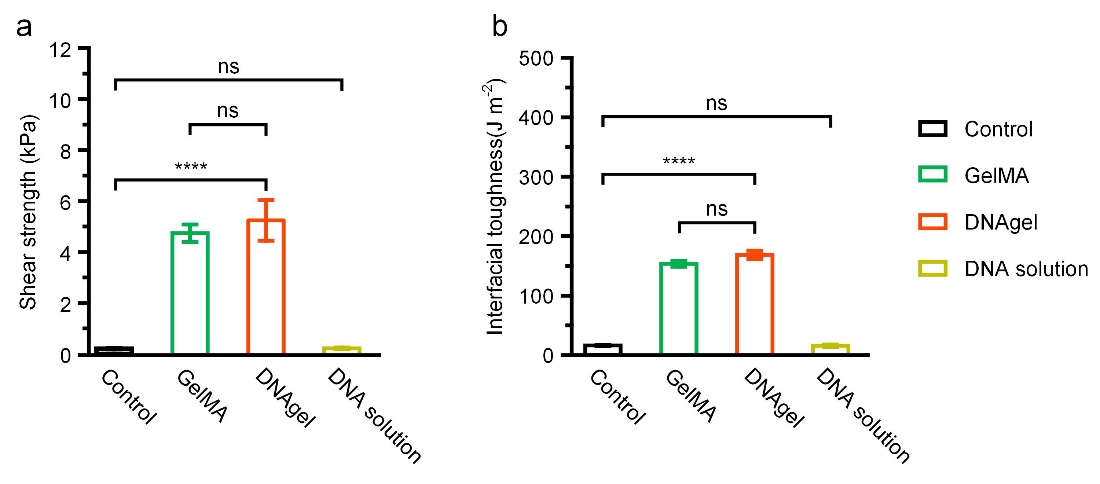


**Supplementary Figure 2.** Wet adhesion to tissue. (**a**) Lap shear tests and (**b**) 90-degree peel tests of different materials with rat dorsal skin (1cm$\times$2cm). Data were presented as mean ± SD (*n* = 3). Statistical analysis was performed by one-way ANOVA with Tukey’s multiple comparisons (**a**, **b**). Source data are provided as a Source Data file.


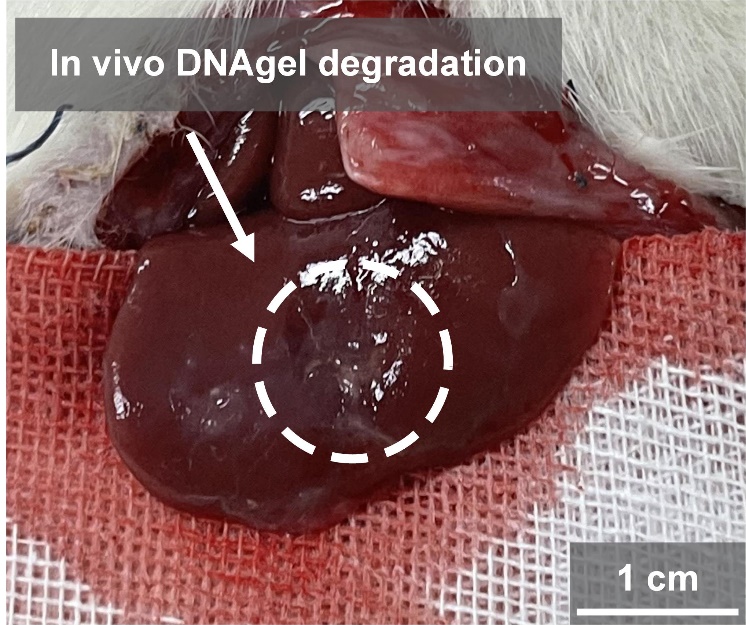


**Supplementary Figure 3.** Representative image of DNAgel degradation in vivo after 7 days. Representative images are shown from three independent experiments with similar results.


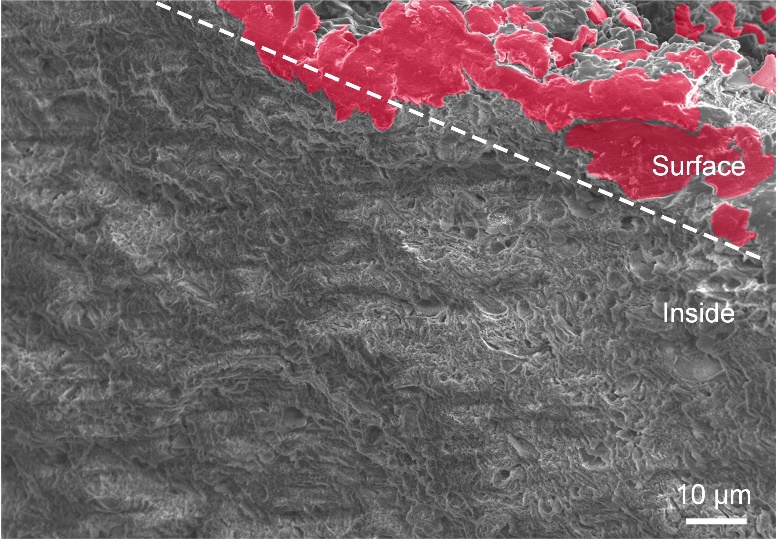


**Supplementary Figure 4.** SEM image of blood cells accumulated on the surface of the DNA hydrogel after *in vivo* hemostatic experiment in femoral artery injury model. Representative images are shown from three independent experiments with similar results.


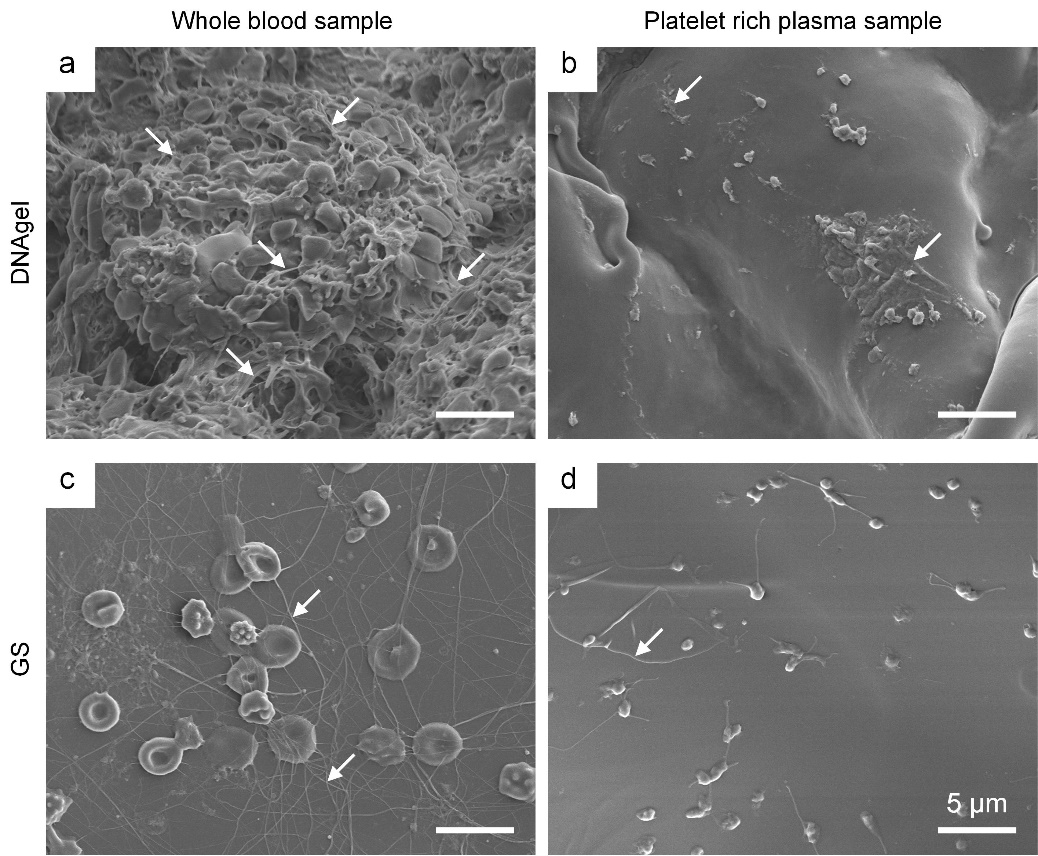


**Supplementary Figure 5**. Representative SEM images of fibrin (white arrow) adhered to the surface of DNAgel and GS. (**a**) DNAgel treated with whole blood, (**b**) DNAgel treated with platelet rich plasma, (**c**) GS treated with whole blood, and (**d**) GS treated with platelet rich plasma. Representative images are shown from three (**a**-**d**) independent experiments with similar results.

**
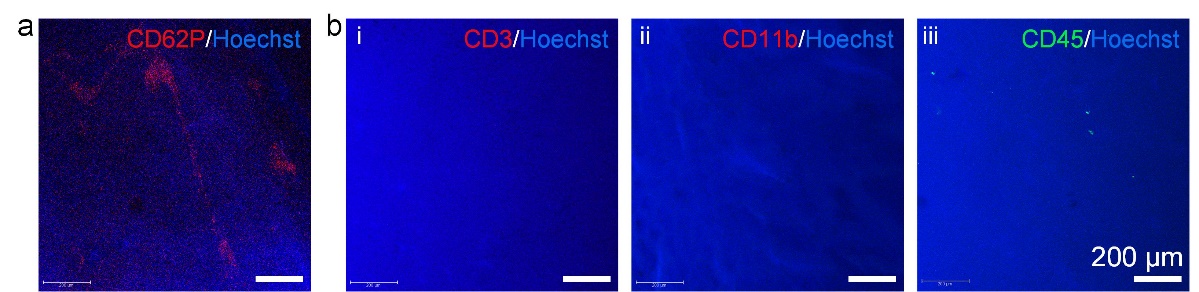
**

**Supplementary Figure 6**. Activated platelets adhered to DNAgel. (**a**) CLSM images of activated platelets (red) aggerated and adhered to the surface of DNA network (blue). (**b**) lymphocytes (CD3), monocytes (CD11b), and leucocytes (CD45) were barely observed on the surface of the DNAgel (blue). Representative images are shown from three (**a**, **b**) independent experiments with similar results.


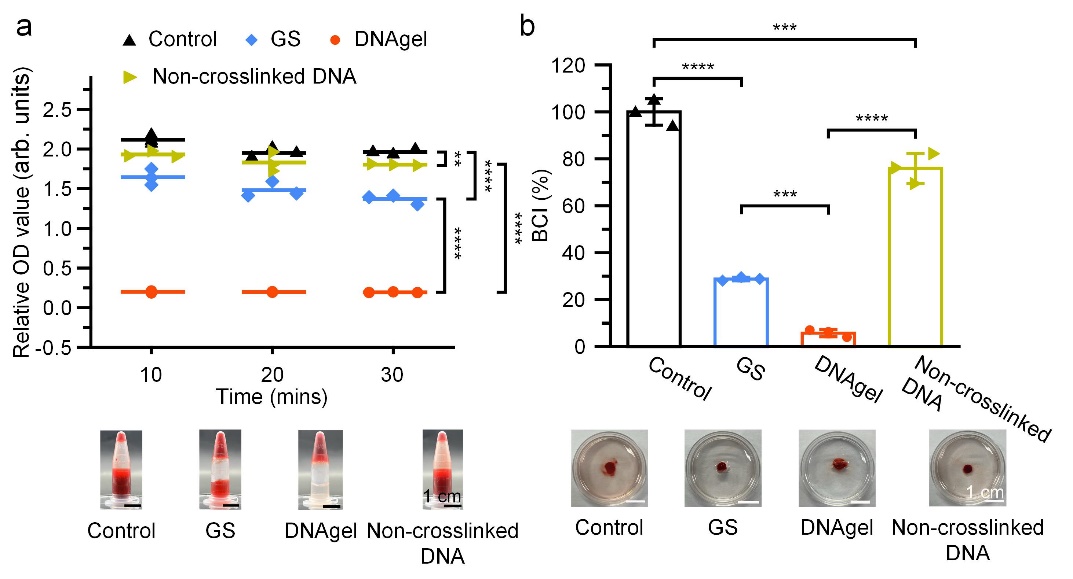


**Supplementary Figure 7.** *In vitro* hemostasis of DNAgel. (**a**) Absorbance of blood clotting samples at different time points measured at 540 nm with UV–vis spectrophotometer and the representative pictures at 30 mins. Scale bar = 1 cm. (**b**) BCI and representative pictures. Scale bar = 1 cm. Data were presented as mean ± SD (*n* = 3). Statistical analysis was performed by one-way ANOVA with Tukey’s multiple comparisons (**a**, **b**). Source data are provided as a Source Data file. Representative images are shown from three (**a**, **b**) independent experiments with similar results.

**
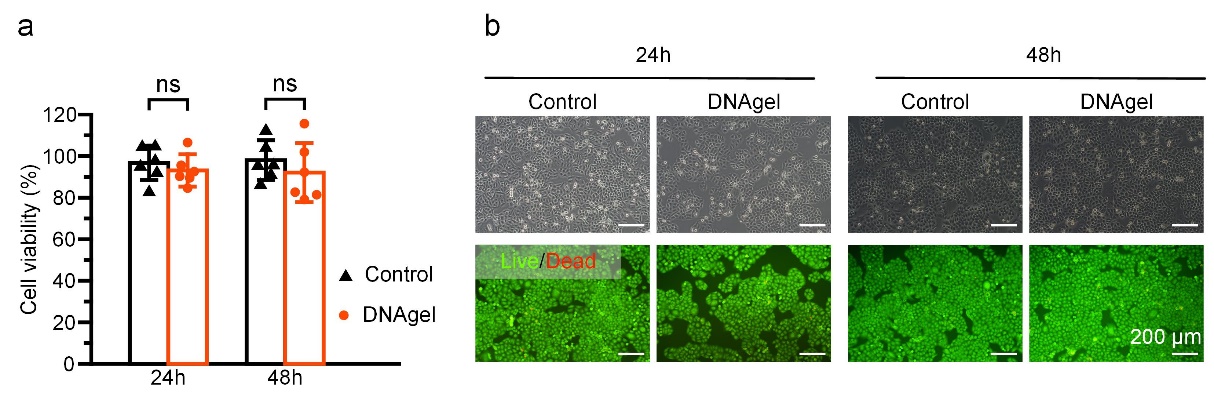
**

**Supplementary Figure 8.** Biocompatibility of DNAgel. (**a**) *In vitro* cell viability and (**b**) representative fluorescent images of WRL68 human normal liver cells incubated with DNAgel-conditioned medium. Scale bar = 200 μm. Data were presented as mean ± SD (*n* = 6). Statistical analysis was performed by unpaired two-tailed Student’s t testing (**a**, **b**). Source data are provided as a Source Data file. Representative images are shown from three (**a**, **b**) independent experiments with similar results.


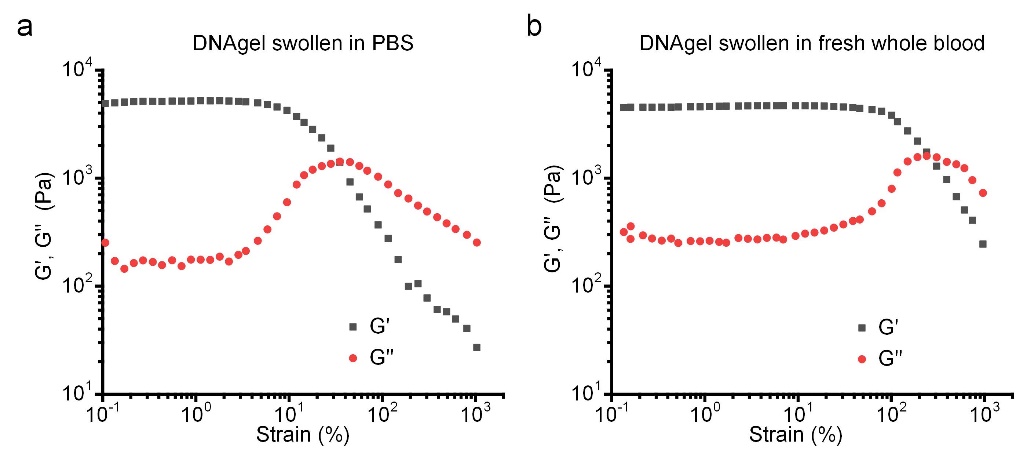


**Supplementary Figure 9.** Oscillatory strain amplitude sweeps of DNAgel. G′ and G″ of DNAgel that well-swollen in (**a**) PBS and (**b**) fresh whole blood on strain amplitude sweep.


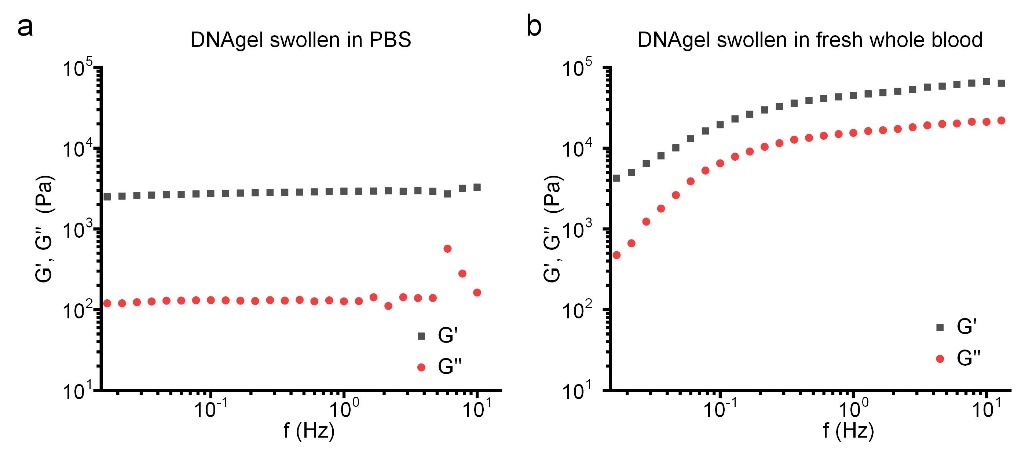


**Supplementary Figure 10.** Oscillatory frequency sweep measurements of DNAgel. G′ and G″ of DNAgel that well-swollen in (**a**) PBS and (**b**) fresh whole blood on frequency sweep.

**
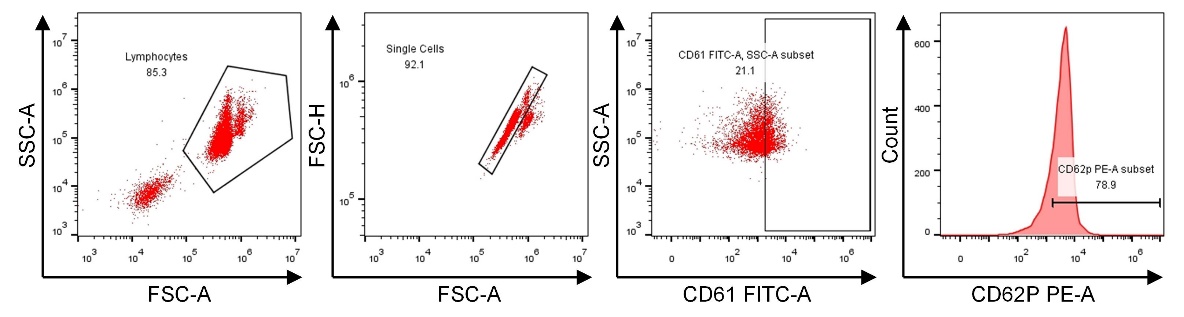
**

**Supplementary Figure 11.** Representative flow cytometry gating strategy for FITC Mouse Anti-Rat CD61 and PE Mouse Anti-Rat CD62P stained cells in whole blood sample with TRAP-6 treated.

**
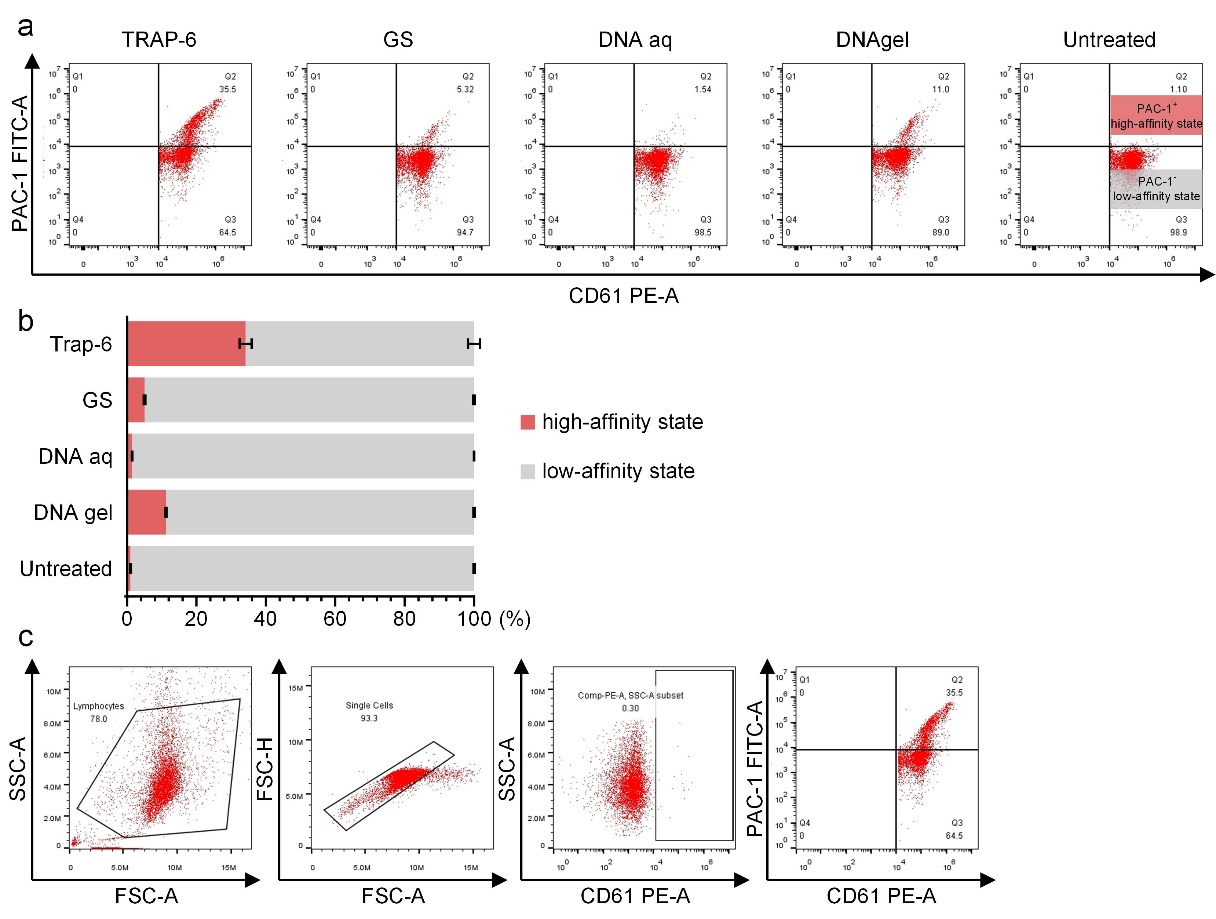
**

**Supplementary Figure 12.** Flow cytometry results of cells stained with CD61 and PAC-1. (**a**) Representative flow cytometry plots of whole blood samples under different treatment conditions. (**b**) Statistical results of GPIIb/IIIa in high-affinity or low- affinity state. Data were presented as mean ± SD (*n* = 3). Source data are provided as a Source Data file. (**c**) Exemplary gating strategy for PE Anti-Human CD61 and FITC Anti-Human PAC-1 stained cells in TRAP-6 treated group.


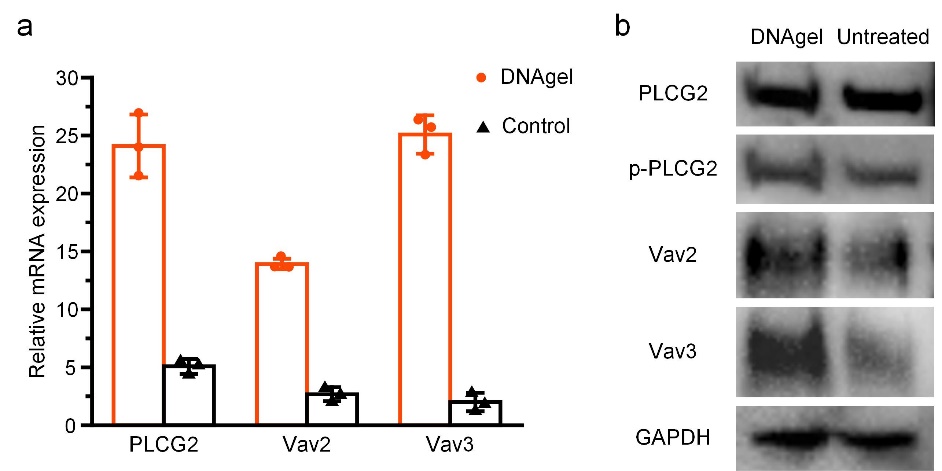


**Supplementary Figure 13**. Changes in related-gene expression under the influence of DNAgel. (**a**) PLCG2, Vav2 and Vav3 mRNA detected in platelets treated with DNAgel. (**b**) Western blot results for the identification of PLCG2, Vav2 and Vav3 protein expression in platelets treated with DNAgel. GAPDH is used as reference protein. Data were presented as mean ± SD (*n* = 3). Statistical analysis was performed by unpaired two-tailed Student’s t testing (**a**). Source data are provided as a Source Data file. Representative images are shown from three (**b**) independent experiments with similar results.

**
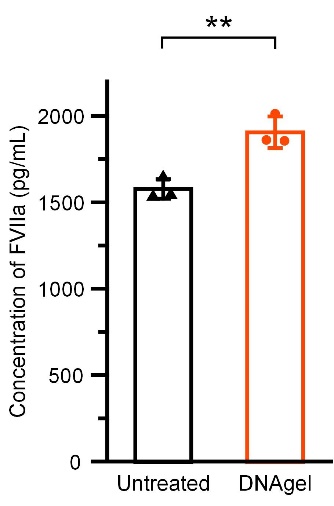
**

**Supplementary Figure 14.** FⅦa content in the DNAgel-treated plasma. Data were presented as mean ± SD (*n* = 3). Statistical analysis was performed by unpaired two-tailed Student’s t testing. Source data are provided as a Source Data file.

**
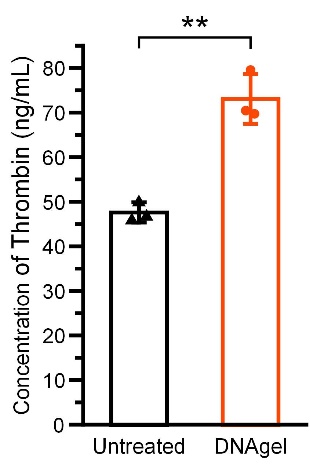
**

**Supplementary Figure 15.** Thrombin content in the DNAgel-treated plasma. Data were presented as mean ± SD (*n* = 3). Statistical analysis was performed by unpaired two-tailed Student’s t testing. Source data are provided as a Source Data file.

**
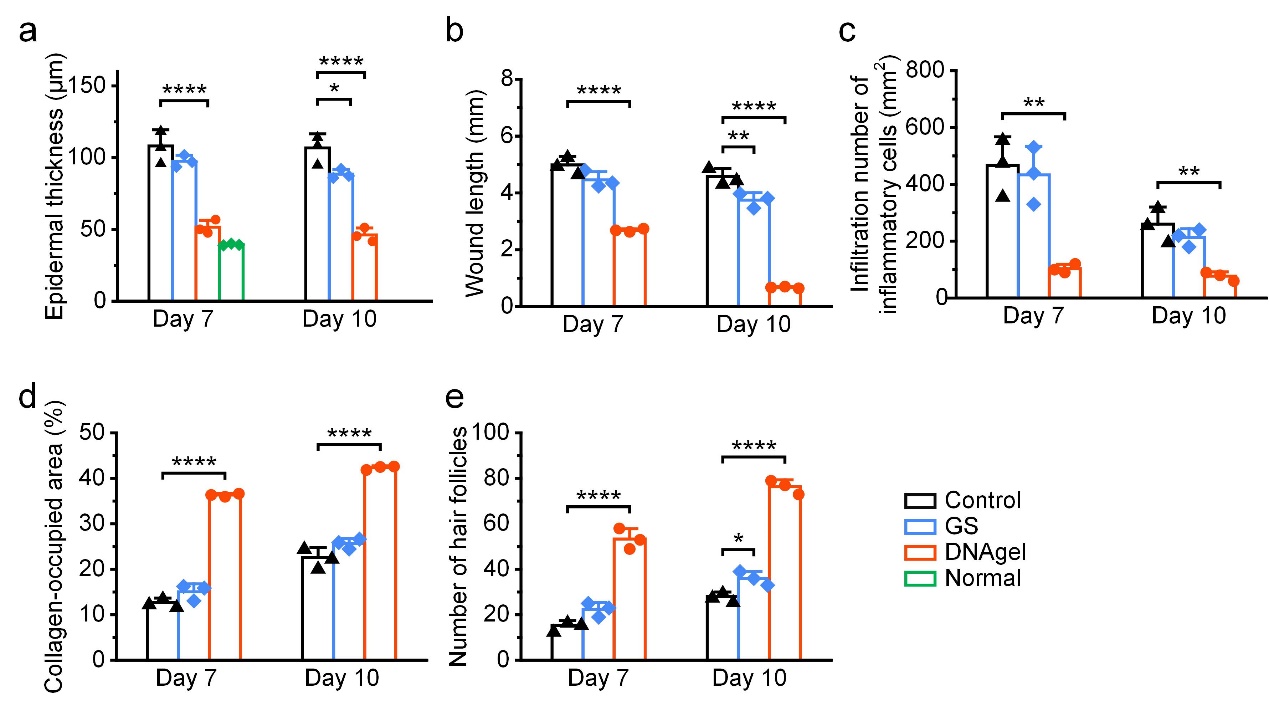
**

**Supplementary Figure 16.** Quantification of histopathologic analysis of each group on Day 7 and 10. (**a**) Epidermal thickness, (**b**) wound length, (**c**) Infiltration numbers of inflammatory cells, (**d**) Collagen-occupied area, (**e**) Number of hair follicles. Data were presented as mean ± SD (*n* = 3). Statistical analysis was performed by one-way ANOVA with Tukey’s multiple comparisons (**a**-**e**). Source data are provided as a Source Data file.

**
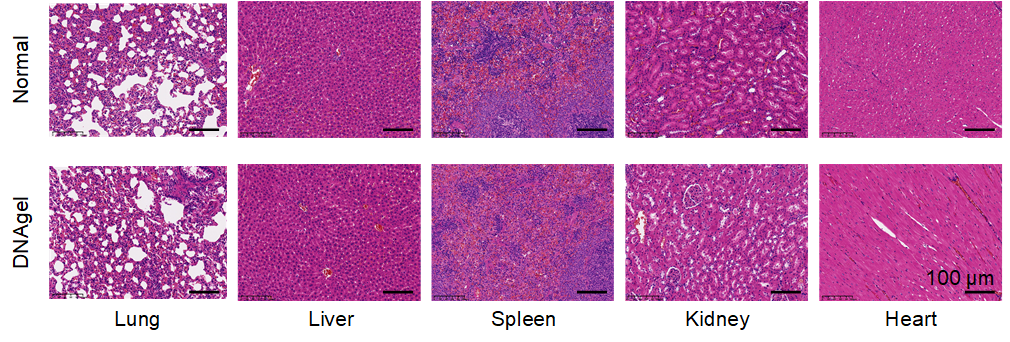
**

**Supplementary Figure 17.** Images of major organ tissue slices with H&E staining in Normal and DNAgel groups. Representative images are shown from three independent experiments with similar results.

**
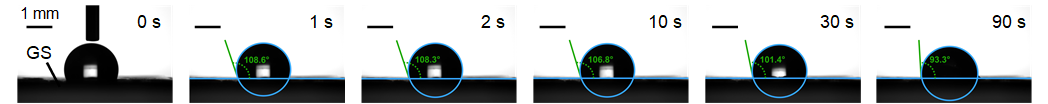
**

**Supplementary Figure 18.** Representative photographs of contact angles for water droplet on Gelatin sponge surface. Representative images are shown from independent experiments with similar results.

**Supplementary Tables**

**Supplementary Table 1:** Sequences of primers used for quantitative polymerase chain reaction (qPCR)

| Primer | Sequence 5’ - 3’ |
| --- | --- |
| PLCG2-F | TCAATGGGCGGACAGGTTA |
| PLCG2-R | CTAGTTTAGGGAGGTGGCGTG |
| VAV2-F | CCATAGTCAACCACACCAAGCA |
| VAV2-R | GAGCATCGACATCGGGAAGGC |
| VAV3-F | CAACCTGAGACCCCAGATGT |
| VAV3-R | TCGGGATAGGCGAGATAATG |

**Supplementary Movies**

**Supplementary Movie 1**. This movie shows the measurement of contact angle for water droplet on DNAgel surface. Speed 5×

**Supplementary Movie 2**. This movie shows the application of DNAgel in rat femoral artery injury model. Speed 2×

**Uncropped scans of blot images of PLCG2, p-PLCG2, Vav2, Vav3, and GAPDH in Supplementary Figure 13**

**
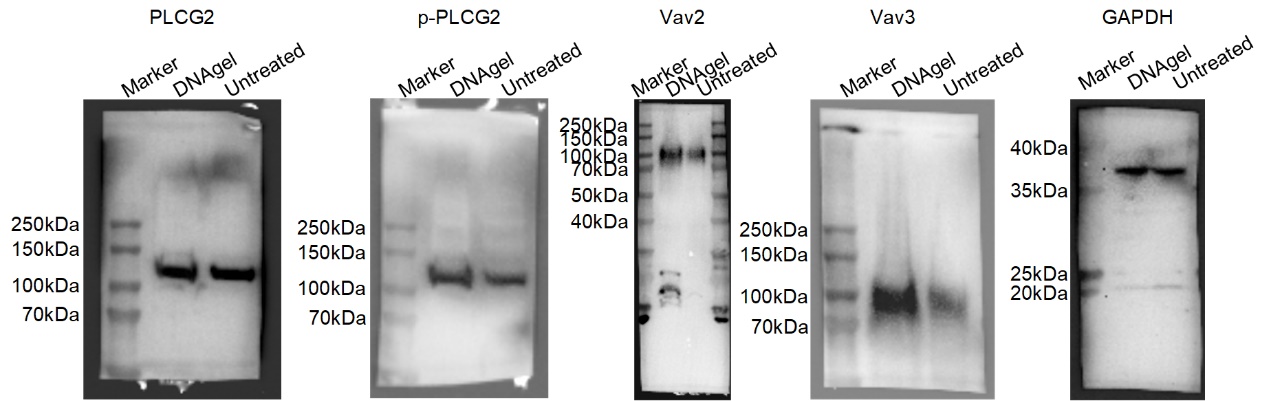
**

**Replicate sample 2**

**
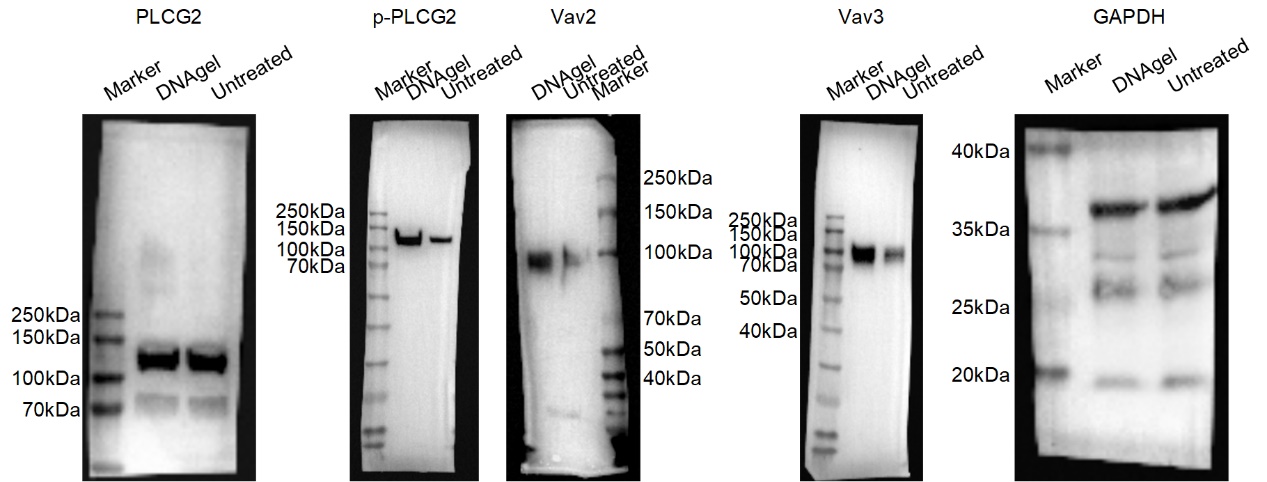
**

**The protein samples were extracted from the biological replicate group of the same experimental protocol.**

**Replicate sample 3**

**
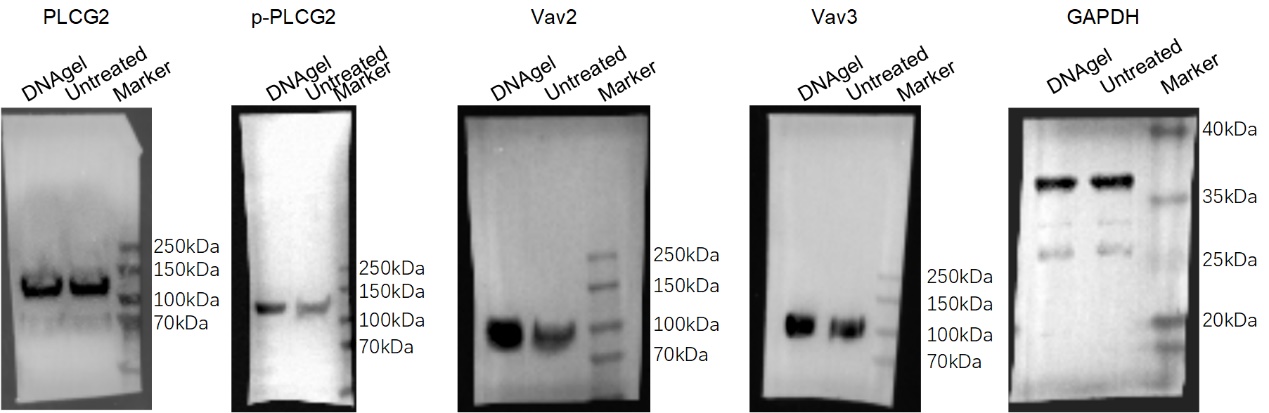
**

**The protein samples were also extracted from the biological replicate group of the same experimental protocol.**

**Standard ladder image of Epizyme, Multicolored Prestained Protein Ladder, Cat no. WJ103**

**
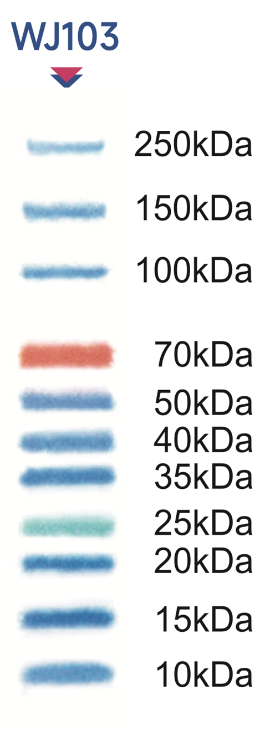
**
