# Supplementary material for: Neutrophil extracellular traps-inspired DNA hydrogel for wound hemostatic adjuvant
Source: Nat Commun. 2024 Jul 2;15:5557. doi: 10.1038/s41467-024-49933-3 (PMC11219873; doi:10.1038/s41467-024-49933-3)
Supplement: Supplementary file 2 — Reporting Summary [file 41467_2024_49933_MOESM2_ESM.pdf]

nature portfolio

Corresponding author(s): *Double-anonymous peer review submissions: write DAPR and your manuscript number here instead of author names.*

Last updated by author(s): *YYYY-MM-DD*

Reporting Summary

Nature Portfolio wishes to improve the reproducibility of the work that we publish. This form provides structure for consistency and transparency in reporting. For further information on Nature Portfolio policies, see our Editorial Policies and the Editorial Policy Checklist.

Statistics

For all statistical analyses, confirm that the following items are present in the figure legend, table legend, main text, or Methods section.

n/a	Confirmed	
☐	☒	The exact sample size (n) for each experimental group/condition, given as a discrete number and unit of measurement
☐	☒	A statement on whether measurements were taken from distinct samples or whether the same sample was measured repeatedly
☐	☒	The statistical test(s) used AND whether they are one- or two-sided<br>*Only common tests should be described solely by name; describe more complex techniques in the Methods section.*
☒	☐	A description of all covariates tested
☐	☒	A description of any assumptions or corrections, such as tests of normality and adjustment for multiple comparisons
☐	☒	A full description of the statistical parameters including central tendency (e.g. means) or other basic estimates (e.g. regression coefficient) AND variation (e.g. standard deviation) or associated estimates of uncertainty (e.g. confidence intervals)
☐	☒	For null hypothesis testing, the test statistic (e.g. F, t, r) with confidence intervals, effect sizes, degrees of freedom and P value noted<br>*Give P values as exact values whenever suitable.*
☒	☐	For Bayesian analysis, information on the choice of priors and Markov chain Monte Carlo settings
☒	☐	For hierarchical and complex designs, identification of the appropriate level for tests and full reporting of outcomes
☒	☐	Estimates of effect sizes (e.g. Cohen's d, Pearson's r), indicating how they were calculated

Our web collection on statistics for biologists contains articles on many of the points above.

Software and code

Policy information about availability of computer code

Data collection	JEOL JSM-IT800, Thermo Scientific HAAKE MARS60, ThermoFisher Nicolet FT-IR, Dataphysics OCA20, Amersham ImageQuant 800, Nikon A1 HD25, MTS CMT6103, Tecan Spark, METTLER TOLEDO ML204T, Veckman Coulter CytoFLEX LX, Leica EM UC7 were used for collecting data.
Data analysis	GraphPad Prism 9.4.1 and OriginPro 2021 were used to perform statistical analysis.

For manuscripts utilizing custom algorithms or software that are central to the research but not yet described in published literature, software must be made available to editors and reviewers. We strongly encourage code deposition in a community repository (e.g. GitHub). See the Nature Portfolio guidelines for submitting code & software for further information.

Data

Policy information about availability of data

All manuscripts must include a data availability statement. This statement should provide the following information, where applicable:

- Accession codes, unique identifiers, or web links for publicly available datasets
- A description of any restrictions on data availability
- For clinical datasets or third party data, please ensure that the statement adheres to our policy

The authors declare that the data supporting the findings of this study are available within the paper and its supplementary information. Source data for each graph and uncropped blot images are provided as a Source Data file. Source data are provided with this paper.

Research involving human participants, their data, or biological material

Policy information about studies with human participants or human data. See also policy information about sex, gender (identity/presentation), and sexual orientation and race, ethnicity and racism.

Reporting on sex and gender	N/A
Reporting on race, ethnicity, or other socially relevant groupings	N/A
Population characteristics	N/A
Recruitment	N/A
Ethics oversight	N/A

Note that full information on the approval of the study protocol must also be provided in the manuscript.

Field-specific reporting

Please select the one below that is the best fit for your research. If you are not sure, read the appropriate sections before making your selection.

[x] Life sciences [ ] Behavioural & social sciences [ ] Ecological, evolutionary & environmental sciences

For a reference copy of the document with all sections, see nature.com/documents/nr-reporting-summary-flat.pdf

Life sciences study design

All studies must disclose on these points even when the disclosure is negative.

Sample size	Animal numbers for each experiment are provided in the figure captions. Each quantitative experiment was performed in at least 3 replicates.
Data exclusions	Abnormal samples, non-experimental related death samples were excluded.
Replication	All experimental findings are reliably reproduced. We have reproduced each experiment at least three times to ensure reliability.
Randomization	All the tests were performed with randomly allocated experimental groups.
Blinding	Experimenters were blinded during life sciences study design. All subsequent sample processing was done blinded.

Reporting for specific materials, systems and methods

We require information from authors about some types of materials, experimental systems and methods used in many studies. Here, indicate whether each material, system or method listed is relevant to your study. If you are not sure if a list item applies to your research, read the appropriate section before selecting a response.

Materials & experimental systems

n/a	Involved in the study	
[ ]	[x]	Antibodies
[ ]	[x]	Eukaryotic cell lines
[x]	[ ]	Palaeontology and archaeology
[ ]	[x]	Animals and other organisms
[x]	[ ]	Clinical data
[x]	[ ]	Dual use research of concern
[x]	[ ]	Plants

Methods

n/a	Involved in the study	
[x]	[ ]	ChIP-seq
[ ]	[x]	Flow cytometry
[x]	[ ]	MRI-based neuroimaging

Antibodies

Antibodies used	Purified Mouse Anti-Human CD61 (BD Biosciences, Cat # 611140)<br>FITC Conjugated Rabbit Anti-Mouse IgG Rabbit Polyclonal Antibody (Huabio, Cat # HA1009)<br>FITC anti-mouse/rat CD61 Antibody (Biolegend, Cat # 104305)<br>PE anti-mouse/rat CD62P (P-selectin) Antibody (Biolegend, Cat # 148305)<br>PE Anti-Human CD61 (Biolegend, Cat # 336405)<br>DUSP2 Monoclonal Antibody (PAC-1), FITC (ThermoFisher, Cat # MA5-28564)

	PLCG2 Monoclonal antibody (Proteintech, Cat # 67011-1-Ig)<br>VAV2 Polyclonal antibody (Proteintech, Cat # 21924-1-AP)<br>VAV3 Polyclonal antibody (Proteintech, Cat # 30291-1-AP)<br>GAPDH Monoclonal antibody (Proteintech, Cat # 60004-1-Ig)
Validation	All antibodies are commercially available and have been tested by the manufacturer, and validation information can be found on the manufacturer's website:<br><br>https://www.bdbiosciences.com/zh-cn/products/reagents/microscopy-imaging-reagents/immunofluorescence-reagents/purified-mouse-anti-human-cd61.611140<br>http://www.huabio.cn/products/Rabbit-Anti-Mouse-IgG-FITC-antibody-HA1009<br>https://www.biolegend.com/en-gb/products/fitc-anti-mouse-rat-cd61-antibody-79<br>https://www.biolegend.com/en-gb/products/pe-anti-mouse-rat-cd62p-p-selectin-antibody-10806<br>https://www.biolegend.com/en-gb/products/pe-anti-human-cd61-antibody-5339<br>https://www.thermofisher.cn/cn/zh/antibody/product/DUSP2-Antibody-clone-PAC-1-Monoclonal/MA5-28564<br>https://www.ptgcn.com/products/PLCG2-Antibody-67011-1-Ig.htm<br>https://www.ptgcn.com/products/VAV2-Antibody-21924-1-AP.htm<br>https://www.ptgcn.com/products/VAV3-Antibody-30291-1-AP.htm<br>https://www.ptgcn.com/products/GAPDH-Antibody-60004-1-Ig.htm

Eukaryotic cell lines

Policy information about cell lines and Sex and Gender in Research

Cell line source(s)	WRL68 cell line was obtained from Procell, Wuhan
Authentication	Cells were used without modification after receiving from the supplier and therefore were not authenticated.
Mycoplasma contamination	All cell lines were tested negative for mycoplasma contamination.
Commonly misidentified lines<br>(See ICLAC register)	No commonly misidentified cell lines were used in the study.

Animals and other research organisms

Policy information about studies involving animals; ARRIVE guidelines recommended for reporting animal research, and Sex and Gender in Research

Laboratory animals	6 week old Sprague-Dawley (SD) rats and C57BL/6 were used in this study.
Wild animals	No wild animals were used in this study.
Reporting on sex	All experimental animals used in this study were male.
Field-collected samples	Samples collected in the field included blood, full-thickness skin tissue
Ethics oversight	All animal experiments were by the Animal Care & Welfare Committee of IBMC (Protocol No. 2022R0015) and Experimental Animal Welfare & Ethical Review Committee of Zhejiang university (Protocol No. ZJU20210268) . This is stated in the manuscript.

Note that full information on the approval of the study protocol must also be provided in the manuscript.

Plants

Seed stocks	N/A
Novel plant genotypes	N/A
Authentication	N/A

Flow Cytometry

Plots

Confirm that:

- [x] The axis labels state the marker and fluorochrome used (e.g. CD4-FITC).
- [x] The axis scales are clearly visible. Include numbers along axes only for bottom left plot of group (a 'group' is an analysis of identical markers).
- [x] All plots are contour plots with outliers or pseudocolor plots.
- [x] A numerical value for number of cells or percentage (with statistics) is provided.

Methodology

Sample preparation	The samples of whole blood or/and platelet-rich plasma were pre-processed by treated with DNAgel, gelatin sponge, DNA aq, TRAP-6 and untreated at room temperature. Cells were collected and incubated with specific fluoresence-labeled antibodies for 30minutes. The intensity of fluorescence was measured by flow cytometer within 1 h.
Instrument	CytoFLEXLX, Beckman Coulter, USA
Software	CytoExpert
Cell population abundance	By stained with antibodies, cells were separated into different parts and cytometry can calculate the proportion of activated platelets. All tests were performed at more than 10000 cells
Gating strategy	CD61 antibody was used to label all platelets, while CD62P or PAC-1 antibody was used to label activated platelets. Samples with no treatment were stained with/without antibodies to determine gate.

- [x] Tick this box to confirm that a figure exemplifying the gating strategy is provided in the Supplementary Information.
