# Supplementary material for: Neutrophil extracellular traps-inspired DNA hydrogel for wound hemostatic adjuvant
Source: Nat Commun. 2024 Jul 2;15:5557. doi: 10.1038/s41467-024-49933-3 (PMC11219873; doi:10.1038/s41467-024-49933-3)
Supplement: Supplementary file 4 — Description of Additional Supplementary Files [file 41467_2024_49933_MOESM4_ESM.pdf]

Supplementary Movie 1:
This movie shows the measurement of contact angle for water droplet on DNAgel surface. Speed 5×.

Supplementary Movie 2:
This movie shows the application of DNAgel in rat femoral artery injury model. Speed 2×.
